# Metabolic engineering of microbes for oligosaccharide and polysaccharide synthesis

**DOI:** 10.1186/1475-2859-5-25

**Published:** 2006-07-21

**Authors:** Anne Ruffing, Rachel Ruizhen Chen

**Affiliations:** 1 School of Chemical and Biomolecular Engineering, Georgia Institute of Technology, Atlanta, Georgia, 30332-0100, USA

## Abstract

Metabolic engineering has recently been embraced as an effective tool for developing whole-cell biocatalysts for oligosaccharide and polysaccharide synthesis. Microbial catalysts now provide a practical means to derive many valuable oligosaccharides, previously inaccessible through other methods, in sufficient quantities to support research and clinical applications. The synthesis process based upon these microbes is scalable as it avoids expensive starting materials. Most impressive is the high product concentrations (up to 188 g/L) achieved through microbe-catalyzed synthesis. The overall cost for selected molecules has been brought to a reasonable range (estimated $ 30–50/g).

Microbial synthesis of oligosaccharides and polysaccharides is a carbon-intensive and energy-intensive process, presenting some unique challenges in metabolic engineering. Unlike nicotinamide cofactors, the required sugar nucleotides are products of multiple interacting pathways, adding significant complexity to the metabolic engineering effort. Besides the challenge of providing the necessary mammalian-originated glycosyltransferases in active form, an adequate uptake of sugar acceptors can be an issue when another sugar is necessary as a carbon and energy source. These challenges are analyzed, and various strategies used to overcome these difficulties are reviewed in this article. Despite the impressive success of the microbial coupling strategy, there is a need to develop a single strain that can achieve at least the same efficiency. Host selection and the manner with which the synthesis interacts with the central metabolism are two important factors in the design of microbial catalysts. Additionally, unlike *in vitro *enzymatic synthesis, product degradation and byproduct formation are challenges of whole-cell systems that require additional engineering. A systematic approach that accounts for various and often conflicting requirements of the synthesis holds the key to deriving an efficient catalyst.

Metabolic engineering strategies applied to selected polysaccharides (hyaluronan, alginate, and exopolysaccharides for food use) are reviewed in this article to highlight the recent progress in this area and similarity to challenges in oligosaccharide synthesis. Many naturally occurring microbes possess highly efficient mechanisms for polysaccharide synthesis. These mechanisms could potentially be engineered into a microbe for oligosaccharide and polysaccharide synthesis with enhanced efficiency.

## Review

Oligosaccharides and polysaccharides are found in nature as components of a broad range of molecular structures, such as cell surface glycoproteins and glycolipids. Carbohydrate moieties of these structures play vital roles in cellular communication processes, as points of attachment for antibodies and other proteins, and as receptors for bacteria and viral particles [1]. Oligosaccharides and polysaccharides therefore have enormous potential as therapeutic agents. However, this potential is far from being realized due to the complex structure of oligosaccharides which makes classical chemical synthesis difficult [2]. Biocatalytic strategies, using either enzymes or whole-cells, represent a realistic and scalable approach to the enormous challenges in synthesizing complex carbohydrates. In this review, we focus on advances made in recent years in developing efficient microbial whole-cell catalysts through metabolic engineering. Due to space limitations, enzymatic approaches, where isolated enzymes are used, are not covered here. Several recent reviews provide excellent overviews of research activities utilizing the enzymatic approach [3-7].

## Oligosaccharide structures synthesized with metabolically engineered microbes

Metabolic engineering of microbial cells represents one of the most promising strategies for deriving oligosaccharides in sufficient quantities to support research and clinical development. Structures synthesized using metabolically engineered microbes reported in literature since 1998 were surveyed and are tabulated in Table 1 (see Additional file 1). Oligosaccharides, di-, tri-, tetra-, and penta-saccharides of diverse structures were successfully synthesized with engineered *Escherichia coli, Pichia pastoris, Agrobacterium *sp. *Corneybacterium ammoniagenes*, and *Corneybacterium glutamicum*. The sugar nucleotide regeneration is central to these metabolic engineering efforts, and many ingenious methods were developed. Depending on the specific sugar nucleotide, the methods vary considerably in the degree of complication. With only one exception (truncated bovine α-1,3-galactosyltransferase), these syntheses exploited microbial glycosyltransferase enzymes, many of which were originated from microbial pathogens. One important advantage of the whole-cell approach is the scalability. Since it avoids expensive starting materials and enzyme isolation, once the strain is developed, it can be easily scaled-up in a fermentor to produce the needed quantity of compounds. This is reflected in the scale of synthesis that has been carried out (up to 2 L, Table 1; see Additional file 1). Product concentration was also impressive, especially in the microbial coupling approach. A concentration as high as 188 g/L (372 mM), highest ever reported for oligosaccharides, was obtained [8].

The following sections detail various metabolic engineering strategies used in providing the necessary elements for synthesis (donors, acceptors, glycosyltransferases). In addition, several unique metabolic engineering challenges in oligosaccharide synthesis are analyzed.

## Donors

Donor (or sugar nucleotide) provision in oligosaccharide synthesis is very challenging. The sheer number of enzymes involved in the synthesis of a sugar nucleotide makes an enzymatic approach less desirable for large-scale synthesis. Consequently, whole-cell catalysts were adopted for their virtue of facile cofactor provisions.

### UDP-glucose

Since UDP-glucose is a precursor in cell wall biosynthesis, all bacteria are capable of its synthesis, at least at a basal level. Although UDP-glucose has not directly been used in oligosaccharide synthesis, its efficient regeneration is important as a precursor for synthesis of other sugar nucleotides such as UDP-galactose and UDP-GA.

### UDP-galactose

**Figure 1:**
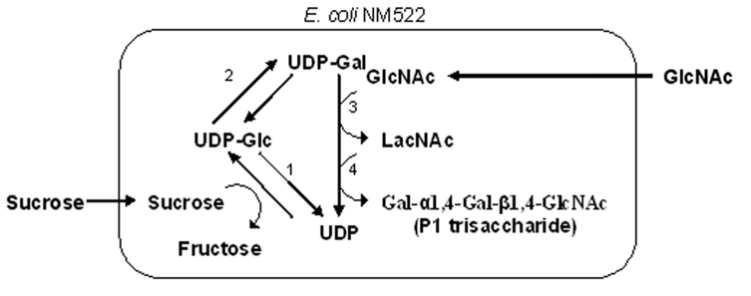
Metabolic pathway for UDP-galactose synthesis using sucrose synthase [9]. Enzymes – 1: sucrose synthase (SusA), 2: UDP-glucose 4'-epimerase (GalE), 3: β1,4-galactosyltransferase (β1,4GalT), 4: α1,4-galactosyltransferase (α1,4GalT).

**Figure 2:**
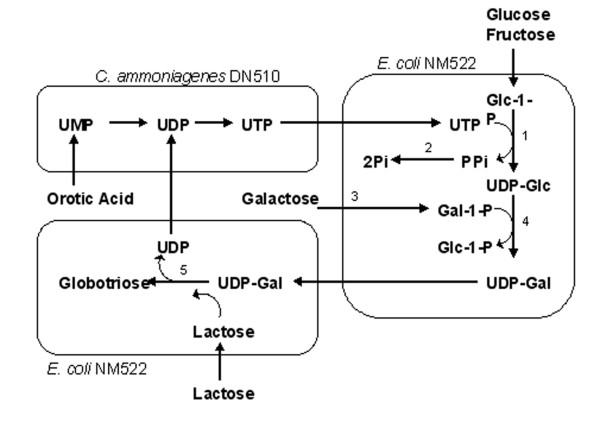
Metabolic pathway for UDP-galactose synthesis using galactokinase and glucose-1-phosphate uridyltransferase [8]. Enzymes – 1: glucose-1-phosphate uridyltransferase (GalU), 2: pyrophosphatase (ppa), 3: galactokinase (GalK), 4: galactose-1-phosphate uridyltransferase (GalT), 5: α1,4-galactosyltransferase (lgtC).

**Figure 3:**
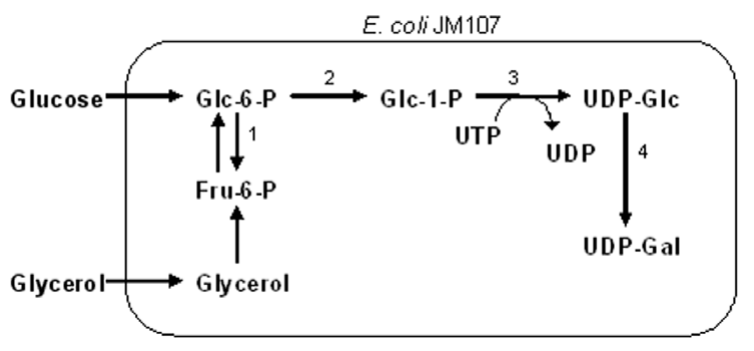
Metabolic pathway for UDP-galactose synthesis using endogenous UDP-glucose synthesis pathway [11]. Enzymes – 1: phosphoglucose isomerase, 2: phosphoglucomutase, 3: UDP-glucose pyrophosphorylase, 4: UDP-glucose 4'-epimerase.

UDP-galactose is by far the best-studied cofactor. Several methods were developed to regenerate this important cofactor. One approach utilizes a sucrose synthase that activates sucrose with UDP, a glycosylation co-product, yielding UDP-glucose. UDP-glucose is then converted to UDP-galactose using a UDP-galactose 4' epimerase (Figure 1). This approach delineates the synthesis from the cell's energy metabolism, since UTP is not required in the cofactor regeneration. Using this method, the product, P1 trisaccharide, could be accumulated up to 50 mM [9]. The degradation of UDP-glucose seemed to prevent further increase of the product, and 5 mM of UDP-glucose was required to start the synthesis. The second approach uses galactose kinase and glucose-1-phosphate uridyltransferase (Figure 2). This route requires the cell to use galactose and another monosaccharide such as fructose to synthesize UDP-galactose, and UTP is needed to regenerate the sugar nucleotide in each cycle of synthesis [8,10]. The third approach is based on *E. coli *cells' endogenous UDP-glucose synthesis pathway [11,12]. UDP-glucose is synthesized from various carbon sources, but most commonly, glucose and glycerol (Figure 3). An introduction of an epimerase will then allow synthesis of UDP-galactose. This route, like the second approach, requires UTP for regeneration of this cofactor.

### CMP-NeuAc

**Figure 4:**
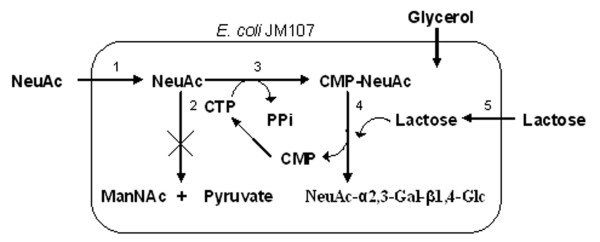
Metabolic pathway for CMP-NeuAc synthesis using CMP-NeuAc synthase [13]. Enzymes – 1: sialic acid permease (NanT), 2: sialic acid aldolase (NanA), 3: CMP-NeuAc synthase, 4: α2,3-sialyltransferase, 5: β-galactoside permease (LacY).

**Figure 5:**
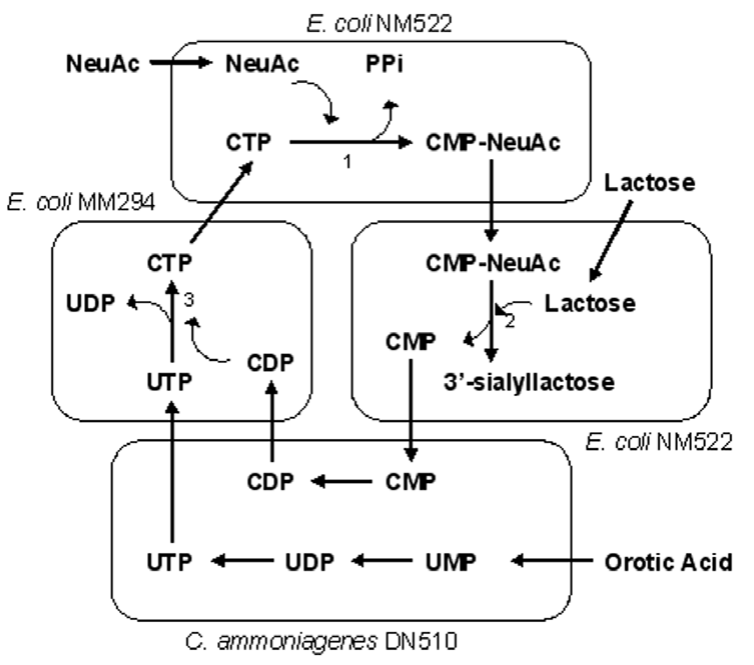
Metabolic pathway for CMP-NeuAc synthesis from NeuAc and orotic acid [14]. Enzymes – 1: CMP-NeuAc synthetase (NeuA), 2: α2,3-sialyltransferase, 3: CTP synthetase (PyrG).

A CMP-NeuAc regeneration system was constructed in an *E. coli *K12 strain by deletion of sialic acid aldolase activity and introduction of CMP-NeuAc synthase (Figure 4). This system requires feeding of sialic acid, thus glucose cannot be used. Furthermore, activation of the sugar relies on the endogenous pool of CTP [13]. This system was successfully used to synthesize sialyllactose, with product accumulation of 1.5 g/L after 22 hours of reaction. In a microbial coupling approach, three bacterial strains were used to synthesize CMP-NeuAc from NeuAc and orotic acid. As shown in Figure 5, the *Corneybacterium ammoniagenes *DN510 was used to supply the high level of UTP needed for CTP synthesis. Additionally, one *E. coli *strain expressing CMP-NeuAc synthetase and another expressing CTP synthetase were required [14]. This system allowed synthesis of 27 mM (17 g/L) CMP-NeuAc, which led to high production of 3'-sialylactose (33 g/L) when coupled to a fourth strain expressing a 2,3-sialyltransferase from *N. gonorrhoeae*.

### GDP-fucose

**Figure 6:**
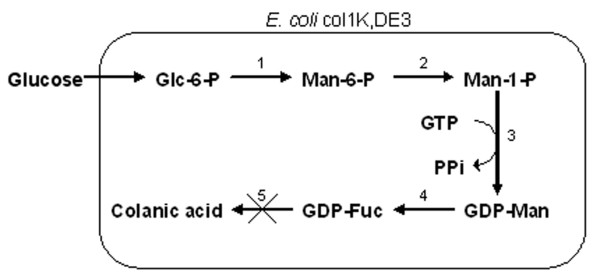
Metabolic pathway for GDP-fucose synthesis using natural GDP-fucose synthesis pathway [15]. Enzymes – 1: phosphomannose isomerase (ManA), 2: phosphomannomutase (ManB), 3: mannose-1-phosphate guanylyltransferase (ManC), 4: GDP-mannose-4,6-dehydratase and GDP-4-keto-6-deoxy-mannose-3,5-epimerase-4-reductase (Gmd, WcaG), 5: putative UDP-glucose lipid carrier transferase (WcaJ).

**Figure 7:**
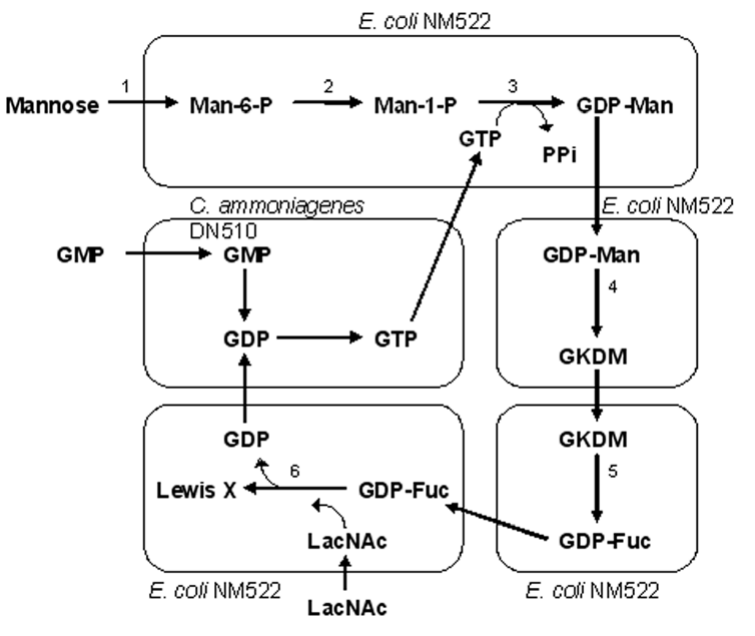
Metabolic pathway for GDP-fucose synthesis using bacterial coupling [16]. Enzymes – 1: glucokinase (Glk), 2: phosphomannomutase (ManB), 3: mannose-1-phosphate guanylyltransferase (ManC), 4: GDP-mannose-4,6-dehydratase (Gmd), 5: GDP-4-keto-6-deoxy-mannose-3,5-epimerase-4-reductase (WcaG), 6: α1,3-fucosyltransferase (FucT).

**Figure 8:**
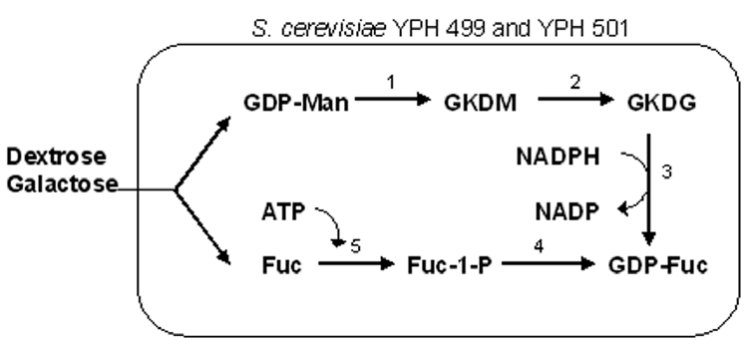
Metabolic pathway for GDP-fucose synthesis in *Saccharomyces cerevisiae *[17]. Enzymes – 1: GDP-mannose-4,6-dehydratase (Gmd), 2: GDP-4-keto-6-deoxy-mannose-3,5-epimerase (GmeR), 3: 4-reductase (GmeR), 4: GDP-fucose pyrophosphorylase, 5: fucose kinase.

One strategy for GDP-fucose production is to enhance the *E. coli *cell's natural synthesis capacity. This was accomplished by overexpressing a positive regulator protein, RcsA, in the colanic acid (a fucose-containing exopolysaccharide) synthesis pathway and by inactivating enzymes involved in GDP-fucose consumption (Figure 6). Collectively, the metabolic engineering strategy re-directs the flux of GDP-fucose destined for colanic acid synthesis to oligosaccharide synthesis. Several fucose-containing products, up to several grams per liter, were obtained with this approach [15]. A bacterial coupling strategy was also developed to produce a high level of GDP-fucose (29 mM, Figure 7). This strategy required four different strains, in which a high level of GTP was produced from GMP with *Corynebacterim ammoniagenes *and precursor, GDP-mannose, was produced from mannose with an *E. coli *strain overexpressing five enzymes involved in the synthesis. Conversion of GDP-mannose to GDP-fucose was accomplished by coupling with two plasmid-bearing *E. coli *NM522 strains expressing GDP-mannose dehydratase and an epimerase/reductase, respectively (Figure 7). Presumably, the number of *E. coli *strains could be reduced to one or two to make the process more efficient. 40 mM (21 g/L) of Lewis X was produced by coupling the GDP-fucose regeneration system with another *E. coli *strain expressing the α1,3-fucosyltransferase from *Helicobacter pylori *[16]. Another approach was developed in *Sacchromyces cerevisiae *by taking advantage of the inherently high mannosylation capability and therefore high level of GDP-D-mannose in this organism. Two *E. coli *genes, encoding GDP-mannose-4,6-dehydratease and a bifunctional GDP-4-keto-6-deoxy-D-mannose-3,5-epmerase/4-reductase, were introduced into the host to endow the yeast strain with the ability to convert GDP-mannose to GDP-fucose (Figure 8). This approach allowed accumulation of 0.2 mg/L (0.35 μM) of GDP-fucose [17]. Although an *in vivo *fucosylation was not demonstrated, it could be expected when combined with a requisite fucosyltransferase.

### UDP-GlcNAc

Like UDP-glucose, UDP-GlcNAc is a precursor to cell wall synthesis. As such, the endogenous UDP-GlcNAc pool can be directly used for oligosaccharide synthesis, and its native synthesis pathway can be used for its regeneration [18].

### UDP-GalNAc

The only report on UDP-GalNAc as a donor in oligosaccharide synthesis was from Samain and coworkers [19]. In this method, the endogenous UDP-GlcNAc was epimerized to UDP-GalNAc using an UDP-GlcNAc 4'-epimerase (WbpP) derived from *Pseudomonas aeruginosa *O6.

Regeneration systems for GDP-Man and UDP-GA have so far not been described in the literature for oligosaccharide synthesis. However, strategies developed for GDP-fucose could also be applied to GDP-Man, a precursor to GDP-fucose. Similarly, a strategy could be devised for regeneration of UDP-GA by introducing UDP-glucose dehydrogenase into the UDP-glucose synthesis pathway.

## Acceptors

A unique problem in oligosaccharide synthesis with metabolically engineered cells is the entry of acceptor sugars. The energy requirement of the synthesis (except in the case of sucrose synthase) necessitates a supply of energy source, most often glucose. Due to selective uptake of this energy source, uptake of the acceptor is not efficient. Several strategies have been developed to improve acceptor uptake. Permeabilization by chemical treatment or freeze-thaw was generally effective in bringing the acceptor sugars (such as lactose and GlcNAc) inside the cells; however, it dissipates the membrane potential required for energy metabolism. Acceptor sugars could be generated inside the cell by introducing suitable enzymes. For example, Samain et al. developed a method to generate chitin oligosaccharide (acceptor sugar) in-situ, which was then further modified to yield chitobiose [18] or was further elaborated through introduction of LgtB to generate a LacNAc moiety [20]. Another method is through the manipulation of the catabolite operon of the acceptor sugars. Significant intracellular accumulation of lactose was achieved by knocking out the *lacZ *gene (encoding β-galactosidase) while leaving *lacY *(lactose permease) intact [13]. An intracellular lactose accumulation of 7–8 mM was observed when the extracellular concentration was 14.6 mM (5 g/L). As a result, lactose could serve as the acceptor for *in vivo *reactions. Several valuable tri-, tetra-, and penta-saccharides were successfully synthesized using this method [13,19].

## Byproduct formation

In *in vitro *enzymatic synthesis, where the supply of donor and acceptor sugars can be conveniently controlled, unwanted byproduct formation is not generally a problem. However, when the synthesis is taking place inside the cell, many byproducts could arise due to a number of reasons. Many glycosyltransferases are promiscuous. LgtB from *Neisseria meningitidis*, for example, can use GlcNAc and glucose, among others, as acceptors, leading to synthesis of LacNAc and lactose, respectively [12]. Although GlcNAc is a slightly favored acceptor as compared to glucose, under *in vivo *conditions, the presence of glucose could lead to a condition that greatly favors the synthesis of lactose. Lactose formation is detrimental to the synthesis process not only causing purification issues, but also draining the UDP-galactose and UTP pools [12], which could otherwise be channeled into LacNAc synthesis.

## Interaction of oligosaccharide synthesis with central metabolism

With the exception of using sucrose synthase for UDP-glucose (and UDP-Gal) regeneration, where the co-product UDP can directly activate the sugar to generate UDP-glucose, oligosaccharide synthesis is intimately dependent upon cellular metabolism. In the case of sucrose synthase, the host is not an active player in the synthesis, it merely provides a milieu in which enzymatic reactions take place. Carbon metabolism is not needed to provide precursors or energy source for the oligosaccharide synthesis. This contrasts with the usual scenario, where a high energy compound such as UTP, GTP, CTP, ATP, or equivalent such as PEP is needed for activation of sugars in each cycle of the reaction. This necessarily brings in the complexity of central metabolism to the oligosaccharide synthesis. Consequently, an energy source is needed, and the carbon flux distributions need to be considered. Since the best synthesis has been demonstrated with bacterial coupling, it is instructive to analyze the energy source and carbon flux for this case. High-energy compounds are produced from orotic acid by a strain efficient in nucleotide synthesis (Figures 2, 5, and 7). The UTP or other equivalents are utilized by the coupled *E. coli *strain(s) engineered to overproduce sugar nucleotides, which in turn are consumed by a strain harboring the necessary glycosyltransferases. This strategy avoids the complication of coupling central metabolism for energy production with the oligosaccharide synthesis by using multiple types of cells, each with a specialized role. Although so far this strategy gives the highest product concentration and provides a realistic technology for commercialization, the requirement of at least three fermentations for a single glycosidic bond makes it a far from ideal solution to a complicated problem. So logically, one asks whether it is possible to achieve the same efficiency in a single strain. From a material balance point of view, the synthesis of an oligosaccharide does not require a constant supply of a nitrogen source, such as orotic acid, if the co-product UDP is truly recycled. It should only require a carbon and energy source to support the synthesis. A single strain strategy could work as efficiently as three, four, or five coupled strains. This assertion comes from the observation of microbial polysaccharide synthesis. Polysaccharide synthesis shares much of the same challenges as oligosaccharide synthesis, being both carbon and energy intensive. Yet successful microbes are found to efficiently channel the carbons to polysaccharides with comparable product yield, product concentration, and productivity to what was reported with the microbial coupling strategy. Examples of those are curdlan-producing *Agrobacterium *sp. and xanthan gum-producing *Xanthomonas campestris*. Therefore, the key to success for a single strain strategy seems to rely on uncovering the secret of polysaccharide synthesis from these microbes, particularly with regard to understanding the coupling of central metabolism to polysaccharide synthesis. The available genome sequences along with proteomic and metablomic tools should facilitate the study toward this goal.

## Host selection

This section only concerns the single strain strategy. A successful oligosaccharide synthesis system, as it becomes clear from the above analysis, requires active glycosyltransferases, an efficient in-situ provision mechanism of donors, internalization of acceptors, and a continuous supply of energy. A host must be able to express the requisite glycosyltransferase enzymes in active forms. Despite impressive successful examples, this is not always the case, especially considering that many interesting glycans are of mammalian-origin. Bacterial glycosyltransferases are extremely useful to avoid the difficulty in expression, but one cannot always find a bacterial version of the glycosyltransferase enzyme required for the synthesis. Therefore, expression of active glycosyltransferases is an important consideration in the choice of a host strain for the metabolic engineering effort. Only a limited number of hosts were used for oligosaccharide synthesis, mainly those that have established genetic tools and commercially available expression vectors.

Donor provision is a tough issue in the engineering process. As reviewed in earlier sections, a strategy to use the cells' natural mechanism for donor regeneration has been successfully used without much genetic manipulation. On the other hand, strategies to create new regeneration mechanisms were also developed. Another interesting strategy has emerged which uses a host that naturally overproduces a sugar polymer. By appropriate engineering, the already efficient mechanism for cofactor regeneration can be harnessed for oligosaccharides synthesis. This strategy was illustrated by Rigngenberg et al. in an *E. coli *strain producing polysialic acid [21]. A null defect in the gene encoding polysialyltransferase (*neuS*) dramatically altered the sialate metabolism, allowing accumulation of CMP-sialic acid to a level more than 50 times higher than that of the wild type. Although this was not demonstrated for sialyl-oligosaccharide synthesis, it is conceivable that by expressing a sialyltransferase and providing a suitable acceptor, the strain could be used for sialyl-oligosaccharide synthesis. In the authors' lab, an *Agrobacterium *sp. strain, naturally overproducing curdlan (a β1,3-glucose homopolymer), was engineered for UDP-galactose regeneration by reducing glucose polymerization. Although the UDP-glucose pool was not significantly elevated by the mutation, oligosaccharide synthesis was much higher than that in an *E. coli *strain under comparable conditions [22]. These examples show that judicious selection of a host strain can lead to an efficient system without overexpression of the enzymes involved in cofactor regeneration. The use of naturally evolved strains for sugar nucleotide regeneration is superior in not only minimizing the genetic modification and resulting metabolic burden of recombinant protein expression, but also ensuring optimal pathway interaction.

Overall, the in-situ provision of donors, acceptor access, and interaction of central metabolism for oligosaccharide synthesis require significant host modification beyond high expression of certain enzymes; therefore, the amenability of the host to genetic engineering is an important consideration in the choice of a host. Broad-host-range expression vectors can be developed for gram-negative bacteria, which allow the introduction of novel enzymes into a wide range of strains, widening the selection of host strains.

## Polysaccharides

The following sections highlight recent metabolic engineering efforts in improving production of sugar polymers. Space constraints make it necessary for us to limit the review to only a few polymers.

### Hyaluronan

Hyaluronic acid (HA) or hyaluronan is a linear polymer of a repeating disaccharide unit, glucuronic acid (GlcA) and *N*-acetyl-glucosamine (GlcNAc). It is found in all vertebrates as a major constituent of the extracellular matrices. In animals, it is found in the vitreous body of the eye, the synovial fluid of articular joints, and the intercellular space of the epidermis. HA is believed to be a lubricant between joint surfaces. It is also involved in morphogenesis and differentiation. Additionally, HA interacts with proteins such as CD44, RHAMM, and fibrinogen, influencing many biological processes such as angiogenesis, cancer, cell motility, wound healing, and cell adhesion [23]. Due to these varied roles, HA influences cell behavior and has significant structural, rheological, physiological, and biological functions in the body. Its distinctive viscoelastic properties, coupled with its lack of immunogenicity and toxicity, have led to a wide range of applications in the cosmetic and medicinal fields, including skin moisturizers, osteoarthritis treatment, ophthalmic surgery, adhesion prevention after abdominal surgery, and wound healing [24]. HA can be either extracted from rooster combs or obtained through fermentation with strains of group C *Streptococcus*. In rooster combs, HA is complexed with proteoglycans, making the isolation of high purity, high molecular weight HA costly [25]. In addition, animal-derived therapeutics carry the risk of transmitting viruses and other adventitious agents as well as inducing immunogenic and inflammatory responses. Due to difficulties in genetic engineering, the *Streptococcus *fermentation route is far from ideal despite its long history as a production strain of HA.

HA is synthesized from two precursors, UDP-glucuronic Acid (UDP-GA) and UDP-*N*-acetylglucosamine (UDP-GlcNAc), by a polymerase called hyaluronan synthase (encoded by *has*A). UDP-GA can be obtained from oxidation of UDP-glucose, which is catalyzed by the NADH-dependent UDP-glucose dehydrogenase. Both UDP-glucose and UDP-GlcNAc are precursors for cell wall biosynthesis. HA synthesis therefore competes with cell growth. Recognizing the high energy demand of HA synthesis, metabolic engineering of *Streptococcus *to improve energy production was attempted by overexpressing NADH oxidase (NOX) and thereby shifting the metabolism from lactate-producing to acetate-producing (energy making) mixed fermentation. This strategy successfully increased the ATP yield by 30%. However, it only led to a 15% biomass increase instead of the desired increase in HA synthesis [26]. This illustrates the difficulty of improving production of a product whose synthesis competes with biomass synthesis in precursor and energy requirements. A successful metabolic engineering strategy will necessarily delineate biomass production from HA synthesis. Other metabolic engineering efforts have been focused on developing suitable microbial production strains. Recently, Novozyme has developed a production system with a *Bacillus subtilis *strain [24]. Although there are no benefits in productivity and HA molecular weight as compared to the *Streptococcus *fermentation, *Bacillus *is a better production strain as it is a GRAS (generally regarded as safe) and the HA produced is not cell-associated, facilitating downstream processing [26]. Another HA synthesis system was developed using chlorella cells infected with Chlorovirus [23]. The yield is about 10 times lower than that of other microbial systems. However, a potential advantage of the system is the higher molecular weight product. This is important as many HA applications are molecular weight dependent. In fact, higher molecular weight HA is highly prized in medical applications such as eye surgery. Derivation of a production strain capable of making high molecular weight HA with mono-dispersity is a holy grail for metabolic engineering efforts. The success of such endeavors awaits a full understanding of the chain termination mechanisms in HA polymer synthesis.

### Alginate

Alginate is a linear copolymer of two-sugar residues, β-D-mannuronic acid (M) and α-L-guluronic acid (G), linked together by 1–4 linkages [27]. The industrially important polymer is produced commercially by extraction from brown seaweeds. Several microbes, such as *Psudomonas aruginosa *and *Azotobacter vinelandii*, naturally produce alginate, and a microbial production method could potentially be developed. As properties of the polymer vary widely with the monomer sugar sequence, microbial systems offer exciting opportunities to produce tailor-made polymers with desired properties. Key to the success of such endeavors is the understanding of enzyme functions involved in alginate synthesis. Of particular interest is the mannuronan C-5-epimerase, which catalyzes post-polymerization conversion of M residues to G residues. Although *Psudomonas aruginosa *encodes only one such enzyme, *Azotobacter vinelandii *has up to 8 epimerases that differ in how the epimerization is introduced, leading to either alternating or G blocks (stretches of consecutive G residues) of different lengths, and thereby considerable diversity of the structure and property. These enzymes have interesting composite structures, containing multiple copies of domains of different functions. This structural feature provides particularly good metabolic engineering targets for rational engineering of polymer properties, provided that the gene-to-enzyme activity relationships are understood in sufficient detail.

### Exopolysaccharides for food use

Exopolysaccharides (EPS) made by bacteria such as *Lactococcus *and *Streptococcus *are an active subject of metabolic engineering. These organisms are widely used in dairy product production. In-situ exopolysaccharide synthesis allows modulation of rheology, improved mouthfeel, and textures of food products. It can also impart some health benefits as prebiotics. However, EPS represents significant metabolic engineering challenges as the natural production level is generally quite low, 50–400 mg/L, when compared to other food-grade polysaccharides such as xanthan (25 g/L, [28]) and curdlan (93 g/L, [29]) made by non-dairy organisms. Metabolic engineering efforts in improving EPS production have been directed to precursor synthesis. Overexpression of UDP-glucose pyrophosphorylase (GalU), an enzyme involved in UDP-glucose synthesis and under the control of a nisin inducible promoter, increased the enzyme specific activity in *Lactococcus lactis *by 20-fold, which in turn increased both UDP-glucose and UDP-galactose synthesis by eight-fold. However, the increased enzyme specific activities did not significantly enhance the synthesis of EPS [30]. Similarly, overexpression of the phosphoglucomutase gene (*pgm*) in *Sphingomonas*, led to a six-fold increase in enzyme specific activity, yet only a small percentage of increase in exopolysaccharides [31]. It appears that, at least in these two examples, the level of precursors was not limiting the EPS synthesis. However, overexpression of both PGM (the enzyme catalyzing the conversion of glucose-6-phosphate to glucose-1-phosphate) and GalU in *Streptococcus thermophilus *led to a two-fold increase in EPS synthesis [32]. Further increase (50%) was obtained when Leloir enzyme (GalK, GalT, and GalU) activities were also increased. While precursor synthesis and interaction with central metabolism are the main focus, other metabolic engineering strategies were also pursued. The increase in the expression of biosynthetic gene cluster in *Sphingomonas *(including glycosyltransferase enzyme activity), for example, improved EPS synthesis by 20% [31]. Further improvement in the production level, an order of magnitude improvement, is desirable for most applications. Therefore, significant challenges still lie ahead. Potentially, not only can the production level be increased, but the composition of EPS could be modified or tailored to target applications.

## Conclusion

Tremendous progress has been made in recent years in complex carbohydrate synthesis. Metabolic engineering has provided the tools for development of highly efficient microbial whole-cell catalysts for oligosaccharide and polysaccharide synthesis. Product concentrations as high as 188 g/L were obtained with engineered strains. An important advantage of using metabolically engineered whole-cell catalyst versus other approaches is the ability to use inexpensive starting material. Consequently, the synthesis could be readily scaled-up according to need. Many valuable oligosaccharides have now been available in gram or larger quantities at realistic costs. An economic analysis puts the cost of oligosaccharide synthesis at $30–50/g [10]. This indeed should be hailed as a significant achievement of metabolic engineering. However, significant challenges still lie ahead. Although the structures synthesized through the engineered strains listed in Table 1 (see Additional file 1) are very impressive, they are only a small fraction of nature's diversity. Many structures of medical importance still await an efficient method for synthesis. For those structures already successfully synthesized, it is important to achieve high efficiency of synthesis in a single strain, as opposed to using coupled microbes, to make the process economically viable.

## Abbreviations

ATP: adenosine triphosphate, CDP: cytidine diphosphate, CMP: cytidine monophosphate, CMP-NeuAc: cytidine monophosphate-*N*-acetylneuraminic acid, CTP: cytidine triphosphate, Fru-6-P: fructose-6-phosphate, Fuc: fucose, Fuc-1-P: fucose-1-phosphate, Gal: galactose, Gal-1-P: galactose-1-phosphate, GDP-Fuc: guanosine diphosphate-fucose, GDP-Man: guanosine diphosphate-mannose, GKDG: guanosine diphosphate-4-keto-6-deoxy-galactose, GKDM: guanosine diphosphate-4-keto-6-deoxy-mannnose, Glc: glucose, Glc-1-P: glucose-1-phosphate, Glc-6-P: glucose-6-phosphate, GlcNAc: *N*-acetylglucosamine, GMP: guanosine monophosphate, GTP: guanosine triphosphate, HA: hyaluronic acid, LacNAc: *N*-acetyllactosamine, Man-1-P: mannose-1-phosphate, Man-6-P: mannose-6-phosphate, ManNAc: *N*-acetylmannosamine, NADP: nicotinamide adenine dinucleotide phosphate (oxidized), NADPH: nicotinamide adenine dinucleotide phosphate (reduced), NeuAc: *N*-acetylneuraminic acid, PEP: phosphoenolpyruvate, Pi: inorganic phosphate, PPi: pyrophosphate, UDP: uridine diphosphate, UDP-GA: uridine diphosphate-glucuronic acid, UDP-Gal: uridine diphosphate-galactose, UDP-GalNAc: uridine diphosphate-*N*-acetylgalactosamine, UDP-Glc: uridine diphosphate-glucose, UDP-GlcNAc: uridine diphosphate-*N-*acetylglucosamine, UMP: uridine monophosphate, UTP: uridine triphosphate.

## Supplementary Material

Additional File 1 **Table 1: **Oligosaccharides synthesized by metabolically engineered microbes from 1998 to present. Table 1 provides a comprehensive list of oligosaccharides synthesized by metabolically engineered microbes from a review of literature since 1998. Click here for file
