# Supplementary material for: Metabolic engineering of microbes for oligosaccharide and polysaccharide synthesis
Source: Microb Cell Fact. 2006 Jul 21;5:25. doi: 10.1186/1475-2859-5-25 (PMC1544344; doi:10.1186/1475-2859-5-25)
Supplement: Additional File 1 — Table 1: Oligosaccharides synthesized by metabolically engineered microbes from 1998 to present. Table 1 provides a comprehensive list of oligosaccharides synthesized by metabolically engineered microbes from a review of literature since 1998. [file 1475-2859-5-25-S1.doc]

|  | **Oligosaccharide** | **Enyzme (glycosyltransferase)** | **Donor** | **Acceptor** | **Host** | **Scale** | **Final Concentration** | **Yield** | **Ref.** |
| --- | --- | --- | --- | --- | --- | --- | --- | --- | --- |
| **Disaccharides** | LacNAc | β1,4-galactosyltransferase (*Helicobacter pylori*) | UDP-Gal | GlcNAc | 2 *E. coli* strains, *C.ammoniagenes* | 30 mL | 157 mM (60 g/L) | 69 % | [33] |
|  | LacNAc | β1,4-galactosyltransferase (*lgtB, Neisseria gonorrhoeae*), | UDP-Gal | GlcNAc | 2 *E. coli* strains,  *C. ammoniagenes* | 2.5 L | 279 mM (107 g/L) | 96 % | [34] |
|  | LacNAc | β1,4galactosyltransferase (*lgtB, Neisseria meningitidis*) | UDP-Gal | GlcNAc, Glc | *E. coli* | 10 mL | 8 mM | 16 % | [12] |
|  | LacNAc | β1,4galactosyltransferase (*lgtB, Neisseria meningitidis*) | UDP-Gal | GlcNAc | *Agrobacterium* sp. | 10 mL | 19.1 mM | 19 % | [22] |
|  | Trehalose (Glcα1,1Glc) | Maltooligosyltrehalose synthase (*treY)*, maltooligosyltrehalose trehalohydrolase (*treZ)*^1^ | N/A | N/A | *C. glutamicum* | 1 L | 9.75 g/L | 49 % | [35] |
|  | Trehalose | *otsBA* operon (*E. coli*) | UDP-Glc (from Glc) | Glc | *C. glutamicum* | 1 L | 10 g/L | 50 % | [36] |
|  | Trehalose | Trehalose-6-phosphate synthase, trehalose-6-phosphate phosphatase (*E. coli* OtsA and OtsB) | UDP-Glc | Glucose-6-phosphate | *C.glutamicum* | 1 L | 3 g/L | 3 % | [37] |
|  | Chitinbiose (GlcNAcβ1,4GlcNAc) | Chitinoligosaccharide synthase (*nodC, Azorhizobium caulinodans*), chitinase (*chiA, Bacillus circulans*) | UDP-GlcNAc | GlcNAc | *E. coli* | 1 L | 4 g/L | 5 % | [18] |
| **Tri-** | Globotriose (Galα1,4Galβ1,4GlcOR) | α-1,4-galactosyltransferase (*Neisseria meningitidis*) | UDP-Gal | Lactose (Galβ1,4Glc) | *E. coli* | 117 mL | 0.92 g/L | 75 % | [10] |
|  | Globotriose (Galα1,4Galβ1,4Glc) | α1,4-galactosyltransferase (*lgtC*, *Neisseria gonorrhoeae*) | UDP-Gal | Gal | 2 *E. coli* strains, *C.ammoniagenes* | 2.5 L | 372 mM (188 g/L) | 79 % | [8] |
|  | Galβ1,4GlcNAcβ1,4GlcNAc | Chitinoligosaccharide synthase (*nodC, Azorhizobium caulinodans*), chitinase (*chiA, Bacillus circulans*), β1,4-galactosyltransferase (*lgtB, Neisseria meningitides*) | UDP-GlcNAc, UDP-Gal | GlcNAc, chitinbiose (GlcNAcβ1,4GlcNAc) | *E. coli* | 1 L | 1.2 g/L | 1 % | [18] |
|  | Galα1,4Galβ1,4GlcNAc | β1,4-galactosyltransferase (*Helicobacter pylori*), α1,4-galactosyltransferase (*Neisseria meningitidis*) | UDP-glucose | GlcNAc | *E. coli* | 200 mL | 50 mM (5.4 g in 200 mL) | 67 % | [9] |
|  | Galα1,3Galβ1,4Glc | truncated bovine α1,3-galactosyltransferase | UDP-Gal | Glc | *P. pastoris* | 200 mL | 28 mM | 37 % | [38] |
|  | GlcNAcβ1,3Galβ1,4Glc | β1,3-N-acetyl glycosaminyltransferase  (*lgtA, Neisseria meningitidis*) | UDP-GlcNAc | Lactose | *E. coli* | 1 L | 6 g/L | 73 % | [13] |
|  | 3’-sialyllactose | α2,3-sialytransferase (*Neisseria gonorrhoeae*) | CMP-NeuAc | Lactose | 3 *E. coli* strains *C.ammoniagenes* | 30 mL, 2 L | 52 mM (33 g/L) | 37 % | [14] |
|  | Sialyllactose (NeuAcα2,3Galβ1,4Glc) | α2,3-sialyltransferase | CMP-NeuAc | Lactose | *E. coli* | 1 L | 1.5 g/L | 16 % | [13] |
|  | α2,3-sialyllactose (and other sialylated oligosaccharides) | CMP-Neu5Ac synthetase (*Neisseria meningitidis*),  α2,3-sialyltransferase (*Neisseria meningitidis*) – fusion protein | CMP-NeuAc | Lactose | *E. coli* | 2.2 L | 67.8 g/L | 68 % | [39] |
|  | Galα1,3LacN_3_ | UDP-galactose 4-epimerase (*galE, E. coli*),  truncated bovine α1,3-galactosyltransferase | UDP-glucose | LacN_3_ | *E. coli* | 5 mL | 0.15-0.17 mM | 60-68 % | [40] |
|  | 3-fucosyllactose (Galβ1,4-[Fucα1,3]Glc) | α1,3-fucosyltransferase  (*futA, Helicobacter pylori*) | GDP-fucose | Lactose | *E. coli* | 1 L | 0.5 g/L (0.95 mM) | 11 % | [41] |
|  | Lewis X (Galβ1,4-[Fucα1,3]GlcNAc) | α1,3-fucosyltransferase (*Helicobacter pylori*) | GDP-fucose (from fucose) | LacNAc | *4 E. coli*  strains *C.ammoniagenes* | 30 mL | 40 mM (21 g/L) | 40 % | [16] |
|  | Lacto-N-neotetraose (Galβ1,4GlcNAcβ1,3Galβ1,4Glc) | β1,3-N-acetyl glycosaminyltransferase  (*lgtA, Neisseria meningitidis*),  β1,4-galactosyltransferase (*lgtB, Neisseria meningitidis*) | UDP-GlcNAc, UDP-Gal | Lactose, GlcNAcβ1,3Galβ1,4Glc | *E. coli* | 1 L | 5 g/L | 47 % | [13] |
| **Tetra-** | GM2 (GalNAcβ1,4-(NeuAcα1,3)Galβ1,4Glc | α2,3-sialyltransferase,  β1,4-GalNAc transferase | CMP-NeuAc,  UDP-GalNAc | Galβ1,4Glc,  NeuAcα1,3Galβ1,4Glc | *E. coli* | 2 L | 1.25 g/L (1.4 mM) | 48 % | [19] |
| **Penta-** | GM1 (Galβ1,3GalNAcβ1,4(NeuAcα1,3)Galβ1,4Glc | α2,3-sialyltransferase,  β1,4-GalNAc transferase,  β1,4-galactosyltransferase | CMP-NeuAc,  UDP-GalNAc,  UDP-Gal | Galβ1,4Glc,  NeuAcα1,3Galβ1,4Glc, GalNAcβ1,4(NeuAcα1,3)Galβ1,4Glc | *E. coli* | 2 L | 0.89 g/L (0.83 mM) | 29 % | [19] |

1. These enzymes do not require a sugar nucleotide donor.
